# Hyperbranched Copolymers of Methacrylic Acid and Lauryl Methacrylate H-P(MAA-co-LMA): Synthetic Aspects and Interactions with Biorelevant Compounds

**DOI:** 10.3390/pharmaceutics15041198

**Published:** 2023-04-09

**Authors:** Anastasia Balafouti, Stergios Pispas

**Affiliations:** Theoretical and Physical Chemistry Institute, National Hellenic Research Foundation, 48 Vassileos Constantinou Ave., 11635 Athens, Greece

**Keywords:** amphiphilic nanoparticles, hyperbranched copolymers, polyelectrolytes, RAFT, hydrophobic drug encapsulation, protein complexes

## Abstract

The synthesis of novel copolymers using one-step reversible addition-fragmentation chain transfer (RAFT) copolymerization of biocompatible methacrylic acid (MAA), lauryl methacrylate (LMA), and difunctional ethylene glycol dimethacrylate (EGDMA) as a branching agent is reported. The obtained amphiphilic hyperbranched H-P(MAA-co-LMA) copolymers are molecularly characterized by size exclusion chromatography (SEC), FTIR, and ^1^H-NMR spectroscopy, and subsequently investigated in terms of their self-assembly behavior in aqueous media. The formation of nanoaggregates of varying size, mass, and homogeneity, depending on the copolymer composition and solution conditions such as concentration or pH variation, is demonstrated by light scattering and spectroscopic techniques. Furthermore, drug encapsulation properties are studied by incorporating the low bioavailability drug, curcumin, in the nano-aggregate hydrophobic domains, which can also act as a bioimaging agent. The interaction of polyelectrolyte MAA units with model proteins is described to examine protein complexation capacity relevant to enzyme immobilization strategies, as well as explore copolymer self-assembly in simulated physiological media. The results confirm that these copolymer nanosystems could provide competent biocarriers for imaging and drug or protein delivery/enzyme immobilization applications.

## 1. Introduction

The design of amphiphilic copolymers for delivery platforms in the field of pharmaceutics and theranostics has presented major progress in the last three decades. Following the mimicry of natural biopolymers such as transport proteins in order to synthesize micellar solubilizing polymer systems, the first in vitro study of sustained drug release from such polymer structures was reported in 1984 [1,2]. Since, many studies of various, commonly referred to as self-assembled, polymeric systems have led to several FDA-approved or even commercially available products to enhance therapeutic pathways [3,4].

Self-assembly is known as a natural phenomenon occurring when molecules with covalently linked hydrophilic and lipophilic compartments are dispersed in aqueous media. In order to achieve thermodynamic stability of the system by reducing the hydrophobic interaction of non-polar groups and water molecules, these molecules conform into core-shell-like structures where the hydrophobic compartments assemble into a core protected by the hydrophilic outer shell [3]. Self-assembled molecules are established as a way to address multiple challenges in the distribution process of many drugs, such as toxicity [5], poor solubility [6] and low bioavailability [7]. Synthetic amphiphilic copolymers have been repeatedly reported to present unique self-assembly behavior, resulting in a variety of polymer nanoparticle (PNP) morphologies and functionalities, which are defined by tunable parameters [8,9,10,11,12]. The advances in controlled/living radical polymerization techniques are a major contribution to this endeavor. Tailoring chemical composition, macromolecular architecture and hydrophilic–lipophilic balance are some of the strategies to regulate self-assembly properties [10,11,12]. 

The influence of macromolecular architecture is an intriguing issue that has been thoroughly studied, especially for linear amphiphilic block copolymers [13,14]. However, recently the interest has shifted towards more complex macromolecular architectures and topologies [15,16]. Hyperbranched copolymers (HCs) are three-dimensional macromolecules composed of highly branched interior structures. Systems of HCs polymers are classified under branched or dendrimer polymers. Excessive random branching of the branch-on-a-branch type, resulting in a highly irregular chain sequence, is the unique characteristic that sets them apart from other branched varieties [17]. Hyperbranched architecture often endows amphiphilic copolymers with better efficiency in the context of delivery applications. For instance, Bej and his group demonstrated the synthesis of amphiphilic hyperbranched polydisulfides that exhibited spontaneous aqueous micellization. The resultant micelles displayed very high non-covalent encapsulation stability towards hydrophobic chromophores while, compared to micelles from their linear analogs, glutathione- (an overexpressed tripeptide in cancer cells able to disrupt disulfide linkages) triggered release was found to be much faster [18]. Several other comparative studies between amphiphilic HCs and their linear counterparts have reported higher loading capacity for intracellular drug delivery [19], improved stability towards coating gold nanoparticles (Au NPs) [20], enhanced thermogelling properties [21], and higher hemocompatibility [22]. Besides the exploitation of the hydrophobic core as a host for guest bioactive compounds, employing the surface of the self-assembled PNPs to attach charged hydrophilic biomolecules or even to improve targeting selectivity and affinity is also a common practice [23,24]. HCs are advantageous in this aspect due to the high density of chain ends (end groups) on the periphery of their spheroid structure [25]. In addition, HCs offer these assets by being cost- and time-effective, since they can be synthesized on a large scale by one-pot reaction [26].

In the present report, we focus on the synthesis of novel amphiphilic poly (methacrylic acid-co-lauryl methacrylate) hyperbranched copolymers, H-P(MAA-co-LMA), and study their self-assembly properties in aqueous media. MAA and LMA are commercially available monomers that have been previously researched as components of biocompatible amphiphilic copolymers [27,28,29,30,31]; however, to our knowledge, their combination has only been reported for linear random copolymers [32,33]. LMA is highly hydrophobic due to the long carbon side chain, whereas MAA is a hydrophilic weak anionic polyelectrolyte, commonly utilized to functionalize the outer shell of self-assembled PNPs with ionizable anionic functional groups [28,29,30]. Two HCs with varying compositions were accessible via reversible addition-fragmentation chain transfer (RAFT) copolymerization [34] of the two monomers of distinctive polarity using a judicial amount of biocompatible difunctional (ethylene glycol dimethacrylate), EGDMA, as a branching agent. Even though combining a reversible-deactivation radical polymerization (RDRP) technique such as RAFT with a multi-functional monomer is a common approach to HCs [17,35], most HCs bearing amphiphilicity are prepared by post-partial hydrophilic or hydrophobic modification of the hyperbranched structure, resulting in homopolymer subunits (block HCs) [36,37,38], while few involve the random distribution of monomers along the HC chains [39,40]. Polymethacrylate particles have been studied as drug delivery systems in the past [41,42]. Usually, systems of methacrylic polymers do not degrade due to the backbone chains being rarely hydrolyzed [43]; however, non-biodegradable polymer products based on MAA or LMA and methacrylic monomers have been previously tested in vitro or in vivo [44,45]. Whilst designing these HCs, we considered biocompatibility as the main criterion to establish an approach for some kinds of applications that do not necessarily require biodegradability. The novel HCs, in terms of composition and architecture, are further studied for their encapsulation and protein complexation or co-assembly properties by utilizing model biorelevant drug curcumin (CUR), and model proteins lysozyme (Lyz) and bovine serum albumin (BSA). Hydrophobic fluorescent drug CUR is specifically utilized in order to describe the potential of drug delivery and bioimaging applications, whereas the interaction with Lyz and BSA reveals protein delivery ability and non-specific protein adsorption of the negatively charged nanoparticles. 

## 2. Materials and Methods

### 2.1. Materials

Monomers methacrylic acid (MAA), lauryl methacrylate (LMA), and ethylene glycol dimethacrylate (EGDMA) were purified using a column that contained butylated hydroxytoluene and hydroquinone monomethyl ether inhibitors removers. 2,2′-azobisisobutyronitrile (AIBN) was recrystallized from methanol. 1,4-dioxane (99.8% pure) was dried through molecular sieves. N-hexane (96% pure), tetrahydrofuran (THF), deuterated tetrahydrofuran (THF-d8), 4-cyano-4-(phenyl-carbonothioylthio)-pentanoic acid (CPAD), pyrene, curcumin (CUR), egg white lysozyme (Lyz), and bovine serum albumin (BSA) were used as such. Except for CUR and EGDMA, which were purchased from Merck and MAA from Acros Organics, all of the materials and chemicals mentioned above were purchased from Sigma-Aldrich (Saint Louis, MO, USA).

### 2.2. Synthesis of H-P(MAA-co-LMA) Copolymers

RAFT polymerization was utilized for the preparation of two H-P(MAA-co-LMA) copolymers with different compositions. In order to achieve the desired macromolecular architecture, a divinyl monomer, EGDMA, was used as a branching agent. AIBN was used as the radical initiator, CPAD as the chain transfer agent and 1,4 dioxane was the selected reaction solvent. The general procedure followed, giving details for HC 1 polymerization reaction as an example, is described below: MAA (1.4 g, 16.3 mmol), LMA (0.6 g, 2.4 mmol), EGDMA (0.076 mL, 0.4 mmol), AIBN (16.421 mg, 0.1 mmol), CPAD (55.876 mg, 0.2 mmol), and 1,4-dioxane (10 mL of total solution) were first placed in a round bottom flask (25 mL) with a magnetic stirring bar. The reaction mixture was then sealed with a rubber septum and deoxygenated for 15 min by nitrogen bubbling. The solution was next placed in an oil bath at 70 °C under stirring. After 24 h of polymerization, the flask was removed from the oil bath and was directly placed at −20 °C until frozen. The still-sealed solution was afterwards brought to room temperature and then unsealed and exposed to air for completion of the polymerization process. Precipitation in excess of hexane was employed as a purification method to remove unreacted monomers or low molecular weight oligomers present in the solution. The participate was finally collected using THF and stored in a refrigerator after drying in a vacuum oven for 48 h. The obtained HCs were later molecularly characterized by SEC, ^1^H-NMR, and FT-IR spectroscopy. Their molecular characteristics are presented in Table 1.

### 2.3. Preparation of Self-Assembled PNPs in Aqueous Solutions

The organic solvent displacement protocol [46] was selected as the general protocol to prepare H-P(MAA-co-LMA) NPs in aqueous media at concentration of 5 × 10^−4^ g/mL. This protocol includes dilution of the solid copolymer in a small quantity of an organic good solvent and addition of the resultant solution into a proper amount of the aqueous medium. The final solution is then heated so that the organic solvent evaporates. In this case, the HCs were initially dissolved in THF overnight. Next, the HC solution was rapidly injected into distilled water under vigorous stirring. Considering the HCs hydrophobicity and specific solubility of MAA, solution pH was manipulated in order to obtain better solubility. Two variations of pH were first obtained, including increasing pH of the aqueous medium before mixing with the HC solution and increasing the pH after mixing with the copolymer and evaporating the organic solvent. The first variation was selected as the general protocol for all sample preparations due to more favorable self-assembly results. Specifically, considering the acidic nature of the HCs, aqueous media with pH 12, using distilled water and NaOH, was previously prepared, so the final HC solution pH was 10. The latter was then used to prepare and study HC solutions of pH 3, 7, and 10, by adding appropriate amounts of HCl. 

### 2.4. Preparation of CUR-Loaded NPs

Samples of 10% weight of CUR compared to copolymer mass were prepared according to the protocol described above. Minor extra steps were taken in order to add CUR to the system. The proper amount of CUR was separately dissolved in THF followed by adding the CUR–THF solution to the THF–copolymer solution. HC concentration was 2.5 × 10^−4^ g/mL and final solution pH was 7. Lower HC concentrations were used so pH was always 7.

### 2.5. Preparation of HC Complexes with Lyz

HCs solutions at pH 7 via the protocol described in Section 2.4 and Lyz stock solutions were separately prepared in distilled water. For each HC, five charge ratios of negatively charged HC to positively charged Lyz were produced. According to the desired HC:Lyz charge ratio, which varied from 2:1 to 1:2, the proper quantity of Lyz solution was rapidly injected into five equal portions of the HC stock solution under stirring. Distilled water was added to the mixed solutions in order to keep HC concentration constant at c = 1.25 × 10^−4^ and 0.75 × 10^−4^ g/mL for all ratios of HC 1 and 2, respectively. Taking into account the hydrophobicity of HC 2, samples of lower concentration were prepared for HC 2.

### 2.6. Preparation of HC–BSA Co-Assemblies

Preparation of 20% and 50% weight of BSA to copolymer mass mixed solutions was similarly obtained by following the steps below. Separate stock solutions of HCs (according to the protocol described in Section 2.3 for pH 7) and BSA (two solutions of different concentration) in distilled water were first prepared. Next, the appropriate amount of BSA solution was rapidly injected into each HC solution under stirring. HC concentration for all samples was 3.08 × 10^−4^ g/mL. The differentiation in the theoretical loading formulation of Lyz and BSA is because Lyz is a cationic protein, and we expect the occurrence of neutralization due to electrostatic interaction of equal negative-to-positive charge ratios. The total negative charge of BSA was not taken into account, since interactions are not expected to occur in the same way. (For an analogous calculation presentation, the %weight of polymer to Lyz at ratio HC:Lyz = 1 is 6.7%).

### 2.7. Experimental Techniques

#### 2.7.1. Size Exclusion Chromatography (SEC)

SEC experiments were conducted on an Agilent 1260 Infinity II HPLC system equipped with one Agilent PolarGel M guard column (particle size = 8 μm) and two Agilent PolarGel M columns (ID = 7.5 mm, L = 300 mm, particle size = 8 μm). Signals were recorded by a UV detector (Agilent 1260 series) and an interferometric refractometer (Agilent 1260 series). The samples were run using DMF with 0.01 M LiBr as the eluent at a temperature of 50 °C and a flow rate of 1.0 mL/min. Molecular weights were determined based on narrow molecular weight linear poly(ethylene oxide) calibration standards.

#### 2.7.2. Proton Nuclear Magnetic Resonance Spectroscopy (^1^H-NMR)

A Varian 300 (300 MHz) spectrometer operated with Vjnmr Software was utilized for ^1^H NMR measurements. HC samples were prepared in THF-d8 at c ≈ 14 mg/mL. Chemical shifts are given in parts per million (ppm) relative to tetramethylsilane (TMS), whereas results were analyzed by MestReNova Software.

#### 2.7.3. Attenuated Total Reflectance–Fourier Transform Infrared (ATR–FTIR) Spectroscopy

FTIR spectra of dry solid copolymer samples were recorded in the region 5000 to 500 cm^−1^ on a Bruker Equinox 55 Fourier transform spectrometer (Bruker, Billerica, MA, USA), which is equipped with a single reflection ATR diamond accessory (Dura-Samp1IR II by SensIR Technologies, Danbury, CT, USA). Spectrums are the result of the average of 64 scans collected at 4 cm^−1^ resolution.

#### 2.7.4. Dynamic Light Scattering (DLS)

DLS studies were performed on an ALV/CGS-3 compact goniometer system (ALV GmbH, Hessen, Germany) equipped with a JDS Uniphase 22 mW He–Ne laser as a light source, operating at 632.8 nm wavelength. An ALV/LSE-5003 light scattering electronics unit was used for stepper motor drive and for limit switch control with an ALV-5000/EPP multi-τ correlator comprising 288 channels. Autocorrelation functions and simultaneously monitored light scattering intensity presented in the results are averaged after five repeated measurements at a goniometer angle of 90°. Results were analyzed by the cumulants method and the CONTIN algorithm. All samples, aqueous and non-aqueous, were filtered through 0.45 μm hydrophilic PVDF filters and hydrophobic PTFE filters, respectively. 

#### 2.7.5. Electrophoretic Light Scattering (ELS)

A Nano Zeta Sizer Malvern instrument equipped with a 4 mW He-Ne laser, operating at 633 nm and a scattering angle of 173°, was used for ELS experiments. Surface charge (zeta potential) values reported were the average of approximately 15 repeated measurements analyzed by the Smoluchowski equation.

#### 2.7.6. Fluorescence Spectroscopy

Fluorescence spectroscopy experiments were conducted on a Fluorolog-3 Jobin Yvon-Spex spectrofluorometer (model GL3-21). Regarding critical aggregation concentration (CAC) determination via pyrene assay, HC solutions in the concentration range of 5 × 10^−4^ to 5 × 10^−9^ g/mL were prepared by successive dilution of a stock solution prepared by following the general solvent displacement protocol, which was mentioned in Section 2.3. Then, a 1 mM pyrene solution in acetone was added to each HC solution at a ratio of 1 μL/mL. The pyrene mixed solutions were left overnight at room temperature in a dark place for the evaporation of acetone. The emission spectra for pyrene were collected in the 355–630 nm range and the excitation wavelength used was λ_ex_ = 335 nm. For protein mixed solution experiments where tryptophan or tyrosine is the fluorescence active residue, the emission spectrum was collected in the range of 310–550 and 290–500 nm, while the excitation wavelength used was λ_ex_ = 290 and 260 nm, respectively. Accordingly, for CUR loading studies, spectra were collected in the spectral region of 435–750 nm and λ_ex_ was 405 nm.

#### 2.7.7. UV-Vis Spectroscopy

Optical absorption spectra of the CUR-loaded NPs were studied in order to investigate drug loading capacity and encapsulation efficiency. The UV-Vis spectra were recorded on a Perkin Elmer Lambda (Waltham, MA, USA) 19 spectrometer between 200 and 800 nm wavelength. An amount of 0.5 mL of the corresponding sample studied by DLS was diluted in 2.5 mL of water or acetone (C_HC_ = 0.42 × 10^−4^ g/mL) for each measurement.

## 3. Results & Discussion

### 3.1. HC Synthesis and Molecular Characterization

**Scheme 1:**
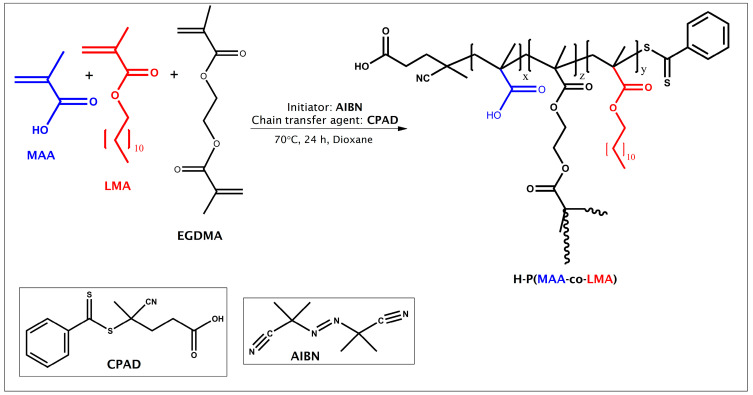
Synthesis route for H-P(MAA-co-LMA) copolymers via RAFT polymerization.

The H-P(MAA-co-LMA) HCs were obtained using one-step RAFT polymerization as illustrated in Scheme 1. The reaction yield was over 94%, while the HCs were received in solid state (Image S1 in Supplementary Materials). The molecular parameters of the reactions and overall molecular characterization results are summarized in Table 1. 

**Figure 1:**
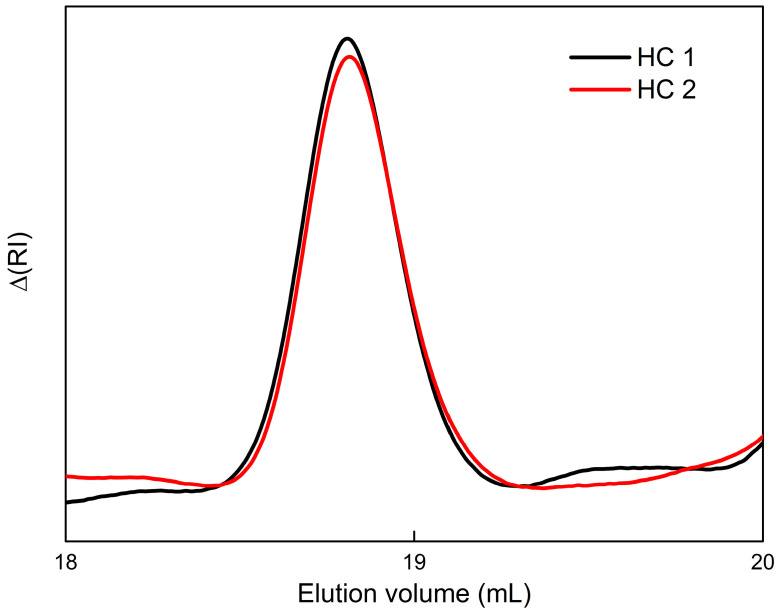
SEC traces of the HCs in DMF.

SEC traces, shown in Figure 1, confirmed the success of the polymerization reaction for both HCs with different compositions. A single symmetric peak, corresponding to narrow molecular weight distributions (polydispersity indexes under 1.3), was observed for both cases. The retention time and consequent values for the apparent number-average molecular weights (M_w_) are attributed to the difference in hydrodynamic volume between the HCs and the linear polymer standards used for calibration. Absolute M_w_ values are estimated to be way higher than the presented apparent ones, which indicates that the copolymers are highly branched. 

**Figure 2:**
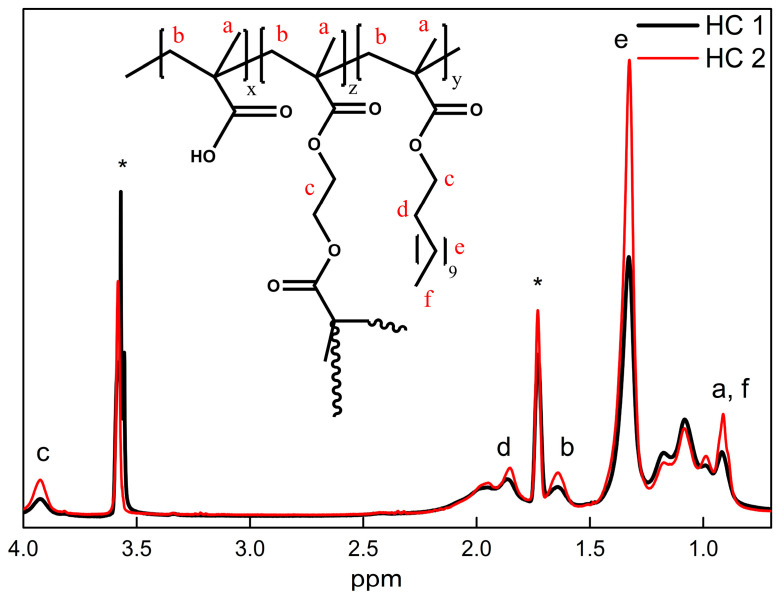
^1^H-NMR spectra of the HCs in THF-d8. (The letters represent peak assignments and the star symbol (*) is attributed to THF-d8).

**Figure 3:**
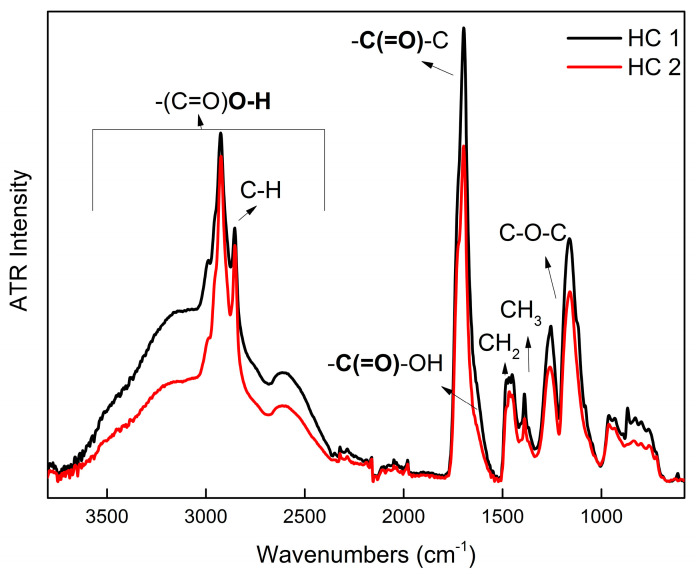
FTIR spectra of the HCs in solid state.

The verification of the chemical structure of the HCs was possible by means of ^1^H NMR and FTIR spectroscopies, while ^1^H NMR spectra, displayed in Figure 2, were also used to calculate the molar ratio between the MAA and LMA units. Since the protons affiliated to the carboxylic acid of the MAA units are usually difficult to detect due to the intermolecular exchange with protons of the solvent, the compositions were calculated by comparing the integration of the signal at δ 1.33 ppm, which correlated to the methylene protons of the LMA pendant chains, and δ 1.64 ppm, which correlated to the methylene protons of the polymer backbone chains [47]. Corresponding integrals are included in Figure S2 of the Supporting Information. The determined composition in the case of HC 2 is very close to the initial monomer feed composition, while in the case of HC 1, a small (11%) positive deviation was observed (Table 1). The chemical composition of the obtained HCs is also translated in their FTIR spectra comparison, which is presented in Figure 3. The characteristic absorption bands at 1470–1370 cm^−1^ assigned to the –CH_2_ and –CH_3_ deformation vibrations display similar intensity, whereas the broad absorption band at 3500–2700 cm^−1^ associated with the –COOH groups appears with clearly higher intensity in the measured spectra of HC 1, indicating the presence of a higher number of MAA units [48]. Additionally, the absence of a clear C=C stretch band in FTIR spectra and the absence of any vinyl proton signal at 6.6–4.0 ppm in ^1^H-NMR spectra, respectively, suggest that monomers, including EGDMA vinyl units, are almost completely consumed [49,50]. The molecular characterization results cooperatively signify control of the polymerization reaction.

### 3.2. Self-Assembly Studies

#### 3.2.1. CAC Determination

**Figure 4:**
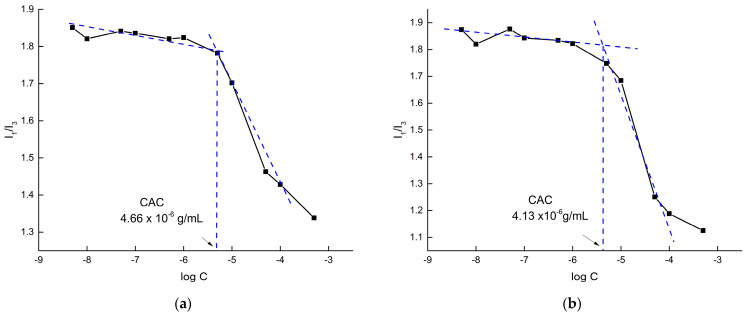
CAC determination for HC 1 (**a**) and HC 2 (**b**) hyperbranched copolymers in pH 7.

The first step to investigate the ability of the HCs to self-assemble into PNPs was a series of fluorescence spectroscopy experiments to determine whether the HCs present a critical aggregation concentration (CAC). At very low concentrations in aqueous solutions, amphiphilic copolymers reside as freely dispersed polymer chains. Increasing the concentration causes an increase in the thermodynamically non-beneficial interactions between hydrophobic moieties and water molecules, forcing the copolymers to self-assemble [51]. CAC or the lowest (critical) concentration in which the copolymers begin to self-assemble provide important information about the copolymers’ system stability and association, since low CAC values foreshadow higher integrity upon extended dilution in the bloodstream [52]. Pyrene, which exhibits characteristic emission spectra that is specifically reflective of the polarity of the micro-environment around it, was utilized as the fluorescent probe. In particular, the ratio of the first peak I_1_ (λ_372_) to the third peak I_3_ (λ_383_) serves as an adequate hydrophobicity scale and therefore indicates the localization of pyrene as free molecules in water (high I_1_/I_3_ values around 1.8) or as entrapped molecules inside the hydrophobic core of the self-assembled structures (low I_1_/I_3_ values around 1.2) [53]. Figure 4 shows the I_1_/I_3_ value plotted against the logarithm of HC concentration. Normally, the point of interception between two tangent lines over the sharp decrease in the I_1_/I_3_ value with the increase in the copolymer concentration is defined as the CAC [53,54].

The HCs studied present low CAC values within a general range of CAC values usually obtained from amphiphilic copolymer systems, typically lower than that of other amphiphiles such as surfactants [55,56]. Moreover, the CAC of HC 2 is slightly lower, while the transition area is clearly sharper. These observations correspond well with the copolymer composition since HC 1, consisting of lower hydrophobic content, is expected to require a higher concentration of polymer chains in order to self-assemble.

#### 3.2.2. Composition Dependency and pH-Sensitivity

**Figure 5:**
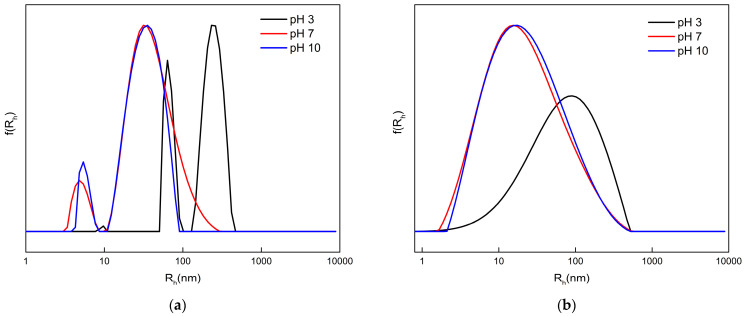
Size distributions from DLS analysis of HC 1 (**a**) and HC 2 (**b**) copolymers in aqueous media in varying pH solutions.

After establishing the self-assembly behavior of the HCs through CAC investigation, DLS experiments were conducted at concentrations above the CAC, in order to acquire details about the size and homogeneity of the PNPs formed. Besides the amphiphilicity and unique macromolecular architecture of the HCs, due to their polyelectrolyte character, electrostatic interactions influence their self-assembly properties as well. DLS studies were hence performed at different pH values for a more complete physicochemical characterization of the assemblies under different solution conditions. Figure 5 shows the size distributions from the DLS analysis of the HCs in solutions of pH 3, 7, and 10, and the results, including mean hydrodynamic radius (R_h_), size polydispersity index (PDI), and scattered light intensity measured at an angle of 90° (I_90°_) for each sample, which are presented in Table 2.

**Figure 6:**
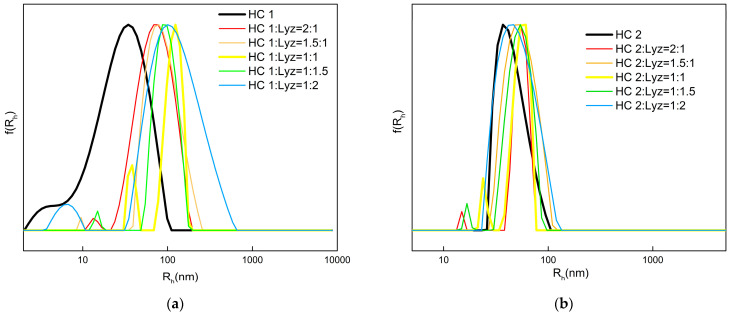
Size distributions of the HC–Lyz complexes formed from HC 1 (**a**) and HC 2 (**b**) copolymers.

Regarding measurements at pH 7, the HCs, in general, form NPs with small sizes of mean R_h_, in the range of 5–40 nm. Judging by the conformation of the HCs in organic solvents such as THF and DMF (good common solvents for both monomer components), which is discussed in the Supporting Information Section (Figure S1, Table S1), it is possible that the population of smaller sizes in the CONTIN histogram of HC 1 constitutes a limited number of uni-molecular self-assemblies. Specifically, a smaller mean R_h_ (in water) than the minimum R_h_ observed when the HCs supposedly exist in the state of freely dispersed polymer chains (in common good solvents) is the lead to this assumption. Another assumption is that these dimensions may represent freely dispersed HC chains with much higher content in the MAA units. This is consistent with the fact this population is only observed for HC 1, of which some chains inherently contain >60% MAA. Moreover, even though HC 2 presents a monomodal size distribution, the relatively high respective PDI in combination with DLS results in lower concentrations (see Figure 6, Table 3), which indicates that an analogous population may exist within the size distribution. 

Either way, both HC 1 and HC 2 present pH-dependent self-assembly. MAA is an anionic polyelectrolyte, with a pK_a_ value of around 4.8. The ionization degree of the pendant acid groups, which depends on the surrounding pH, affects solubility and therefore chain conformation of the hyperbranched polyelectrolyte. Increasing the pH causes an increase in ionization, which usually leads to the swelling of the outer shell of micelle-type structures containing MAA [57]. On the contrary, when the medium pH is lower than the pK_a_, the polymer chains typically shrink and collapse. The HC systems conform to these expectations. In the pH 3 solution, where complete protonation of -COOH is expected, both HCs display an important increase in the mean R_h_, whereas, for HC 1, scattered intensity (I) is significantly greater than the I measured in the pH 7 solution. For HC 2 copolymer, precipitation signs were optically observed, causing a great decrease in the measured I value. Consequently, for both systems, unstable NPs of higher size and mass were observed. This is attributed to the disappearance of electrostatic repulsion forces and the simultaneous dominance of hydrophobic interactions within the HC assemblies [58]. Hence, the collapse phenomenon was observed more intensively for HC 2, where the chains possess higher hydrophobic content. Interestingly, comparing results for pH 7 and 10, there is no clear pattern of swelling with the increase in pH. Decreasing the pH from 10 to 7 causes HC 1 to assemble in slightly looser NPs of larger size (R_h_) and lower mass (lower I). This could be attributed to the further aggregation of some already formed NPs with smaller sizes. However, the alterations are very small in magnitude. The macromolecular hyperbranched architecture and related steric hindrance phenomena could also act as antagonizing factors to the swelling/deswelling processes. More expectedly, HC 2 solely presents an appreciable increase in the measured I, while the size of the NPs remains the same, most likely meaning that, due to more hydrophobic interactions, the system transits to a smaller number of more compact (denser) NPs. 

ELS experiments were performed to assess the surface charge of the NPs formed in each case. The obtained zeta potential (ζ_p_) values shown in Table 2 are in accordance with the composition and relative ionization extent of each copolymer.

### 3.3. Lyz Complexation Studies

The surface charge of HC NPs at pH 7, in theory, provides the functionality for efficient binding with an oppositely charged protein via electrostatic interactions, forming copolymer–protein complexes. Lyz is a well-studied, bioactive, thermostable protein known for its effective antibacterial properties. It is a small-size cationic molecule with a total positive charge of +8 at pH values under the isoelectric point (pI = 10.7); thus, it was selected as a model compound to investigate the polyelectrolyte protein complexation ability of HCs [59,60,61]. Five different negative-to-positive charge ratios between the copolymer and Lyz were studied for each HC, while the HC concentration was kept constant. DLS and ELS measurements of all complexes at different HC:Lyz ratios, and for each HC, are analyzed in Table 3 and the corresponding size distribution graphs from CONTIN analysis are presented in Figure 6.

With respect to the DLS results, for both series of complexes of HC 1 and HC 2, it was observed that the binary mixtures present larger mean R_h_ and I values in comparison with the non-complexed HC solution. Since no mixed sample displays a bimodal size distribution where one of the populations is reminiscent of the non-complexed HC size distribution, it is safe to say that the result above indicates the formation of HC–Lyz complexes. The phenomena are more obvious for HC 1, possibly due to a higher HC concentration in the solution as well as the higher percentage in MAA units. In particular, for HC 1, decreasing the HC:Lyz ratio causes an increase in the mean R_h_, while there is also a linear decrease in the I value. A possible explanation for the difference in measured I is that, from ratio HC:Lyz = 2:1 to HC:Lyz = 1:1 where a theoretically neutralized system occurs [62], some of the Lyz molecules interact within the cavities of already formed loose aggregates of the copolymer hyperbranched chains. Moreover, from the ratio HC:Lyz = 1:1 to HC:Lyz = 1:2, the addition of an excess of Lyz molecules in the system leads to a random arrangement of globular protein molecules on the surface of the polymer aggregates, hence the increase in the PDI value. In the latter case, some bridging effects between complexes may take place (with outer Lyz molecules acting as bridging sites between complexes) and thus increasing the size polydispersity of the species in solution. It is difficult to compare the two HC systems, since complexation is strongly dependent on the original self-assembly of the HCs, which is not similar. HC 2, however, presents a more common behavior, as shown by the measured I value and mean R_h_ peak at ratio HC:Lyz = 1:1, where the highest mass of copolymer/protein complexes is supposed to be observed. It is important to note that, in general, complexation forces the system to somehow better the (size) homogeneity. Additionally, comparing the ζ_p_ values of the systems before and after mixing with Lyz, positive and increased values were observed for the complexes, further confirming complexation via strong electrostatic interactions and the location of Lyz molecules in the outer domain of the complexes (one should take into account that the ζ_p_ value of free Lyz solution at the maximum concentration where Lyz could exist in the samples was around +3 mV). In other words, the ζ_p_ values for the complexes denote that complexation occurs mostly through the decoration of the surface of the PNPs and not through the encapsulation of Lyz inside their cavities.

**Figure 7:**
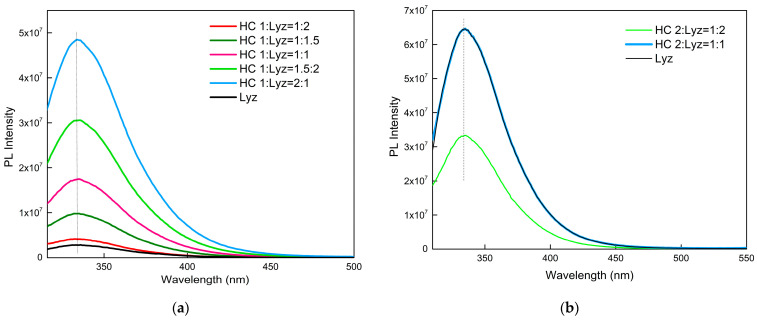
Fluorescence spectra of HC–Lyz complexes of (**a**) HC 1 and (**b**) HC 2.

Fluorescence spectroscopy experiments were performed to examine whether the complexed Lyz presents any conformational changes, which could degrade its biorelevant properties. The investigation was based on the characteristic spectra of tryptophan amino acid residues, which are commonly utilized to inspect the protein chemical environment or any denaturation signs [63]. The spectra for selected samples in comparison to free non-complexed Lyz are given in Figure 7. All samples presented similar spectra, with varying intensity depending on the Lyz concentration, while no shift of the emission maximum wavelength (λ_max_ = 335 nm) was observed, meaning that Lyz preserves its natural conformation and biological properties.

### 3.4. CUR Loading Study

**Figure 8:**
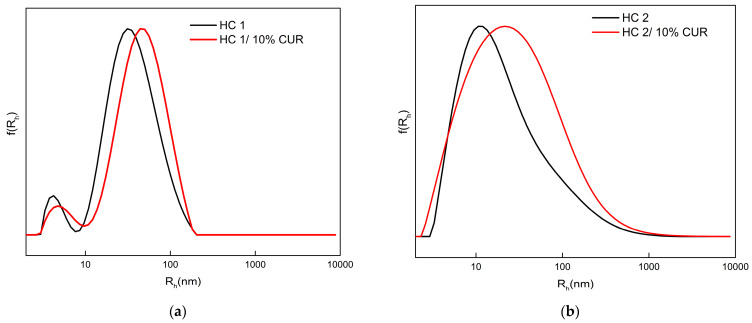
Size distributions from DLS analysis for the neat and CUR-loaded NPs of HC 1 (**a**) and HC 2 (**b**).

The hydrophobic drug CUR was utilized to examine the ability of the HCs to incorporate bioactive compounds that exhibit poor solubility in aqueous media. CUR is well-known for its pharmacological properties, which include molecular-level anti-tumor, anti-inflammatory and antioxidant activity. Moreover, because of its strong natural fluorescence, it is used to investigate the capacity of amphiphilic PNPs to serve as nanocarriers for drug delivery or bioimaging applications [64,65]. The targeted loading level relative to the mass of the HC was 10%. The resultant transparent solutions with the characteristic yellow color of CUR are depicted in Image S2 of the Supplementary Materials. The size distributions from the DLS analysis for the neat and CUR-loaded HC NPs at the same concentration are shown in Figure 8 and the respective DLS and ELS results of the measured samples are presented in Table 4.

Comparing the self-assembly behavior before and after CUR incorporation, it was observed that both systems of HC 1 and HC 2 copolymers produce NPs slightly larger and higher in mass after CUR encapsulation. Since the samples did not display any sign of precipitation, apparently CUR was successfully encapsulated in the hydrophobic core domains of the NPs, causing minimal alterations in their self-assembly behavior. The similarity of the size distribution curves also signifies a relative reproducibility for the systems, which was also pronounced in the samples with complexed Lyz. With respect to ELS measurements, a small increase in the negative surface charge is also coherent to CUR encapsulation. CUR disturbs the hydrophilic–lipophilic balance and, as a result, a larger number of available MAA hydrophilic units may, as of now, reside closer to the surface of the NPs in order to better shield the hydrophobic compartments.

**Figure 9:**
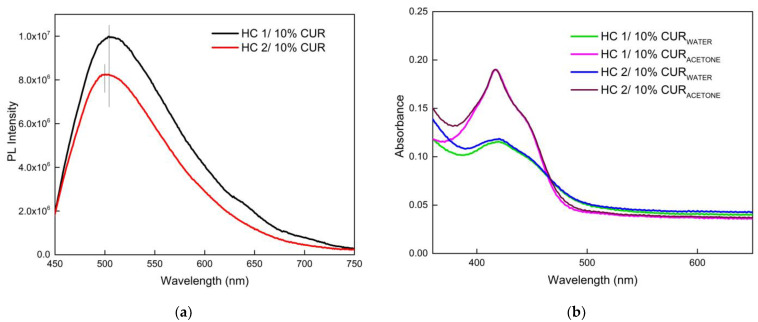
Fluorescence (**a**) and Uv-Vis (**b**) spectra of the CUR-loaded PNPs.

Complementary fluorescence and UV-Vis experiments were performed to further evaluate the encapsulation ability of the HCs. The UV-Vis spectra of diluted HC 1/10% CUR and HC 2/10% CUR samples in acetone, shown in Figure 9b, were used to calculate the drug-loading content (DLC) and drug-loading efficiency (DLE) (Supporting Information). The HCs presented satisfactory values (DLE > 15%), which, unexpectedly, were identical despite the difference in hydrophobic–hydrophilic balance. The characteristic broad absorbance band around 420 nm [66], which is of equal intensity for the two HCs, increases upon dilution in acetone and therefore disruption of the assemblies occurs, indicating that CUR is indeed entrapped into the PNPs, and not able to completely express its optical properties. The solubility of encapsulated CUR was measured as 4.35 μg/mL, at least 300 times the stated CUR water solubility, which is 11 ng/mL [67]. Interestingly, in contrast to UV-Vis spectra, fluorescence spectra (Figure 9a) of the CUR-loaded NPs show some small variations in intensity and emission maximum wavelength, attributed to possible hydrophobic interactions between CUR and LMA segments or the self-quenching phenomena. In general, CUR spectra blue shifts from the reported broad peak around 540 nm in water, reflecting an apolar hydrophobic environment inside the self-assembled aggregate cores [68]. Notably, the more hydrophobic HC 2 displays a somewhat greater shift but lower intensity.

### 3.5. HC–BSA Co-Assembly Studies

**Figure 10:**
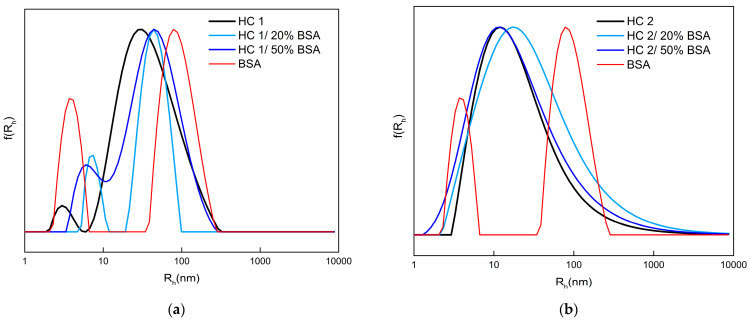
Size distributions from DLS for HC 1 (**a**) and HC 2 (**b**) co-assembly with BSA.

HC co-assemblies with BSA were prepared in order to assess their self-assembly behavior in simulated physiological media and gain information on non-specific protein adsorption on the HC nanoparticles. Furthermore, it is interesting to examine the interaction of HC NPs with a model amphiphilic protein bearing both hydrophobic and hydrophilic moieties as well as a larger number of both positive and negative charges [62]. Figure 10 shows the size distributions of HC–BSA co-assemblies from DLS, comparing neat HC and BSA with two formulations of different HC-to-protein ratios. Adding BSA to the HC systems causes a very small shift of the size distribution curves towards larger sizes and higher mass (slight increase in the measured I value) NPs (Table 5). The self-assemblies of the HCs are less influenced by the BSA, as observed by the disappearance of a large fragment of the population of neat BSA. Due to higher total hydrophilicity, the protein is possibly more flexible to adjustments that are forced either by hydrophobic or electrostatic interactions after mixing with copolymer aggregates. At physiological pH, BSA possesses eight anionic charges; therefore, because of repulsive forces between the latter and the polyanion MAA units on the self-assembled PNPs, electrostatic complexation is not favored. However, studies of amphiphilic polyanions and BSA co-assemblies have shown that binding takes place through hydrophobic and hydrogen bonding interactions or through binding to cationic patches on the surface of protein. Specifically, the surface tension and hydrogen bonding interaction of the HC carboxyl and BSA amino group would contribute to this [69,70]. The ζ_p_ values (Table 5) of the co-assemblies are also closer to the values of neat copolymer and higher than the expected values for the mixture of the two solutions. This might imply that some positive surface charges, probably exposed by the conformation of BSA, are included, contributing to the copolymer–protein interactions.

**Figure 11:**
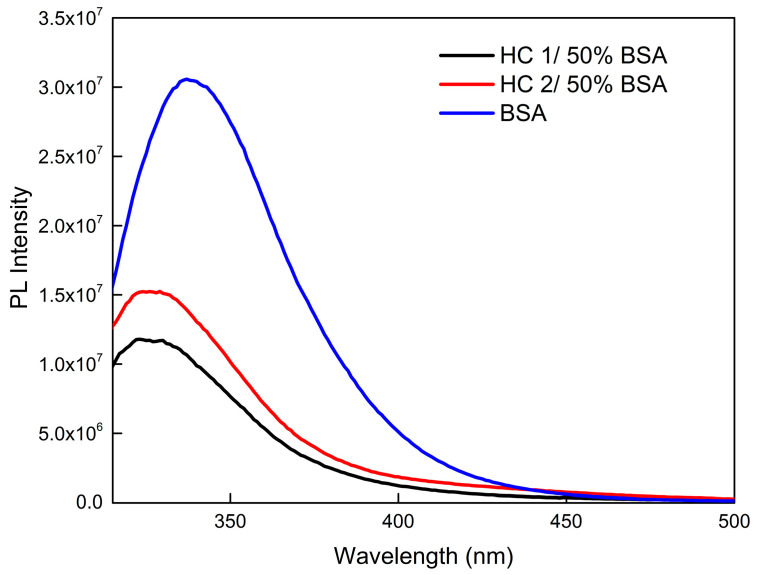
Fluorescence spectra of selected HC–BSA co-assemblies.

BSA contains two tryptophan residues, where one is located within a hydrophobic pocket and the other is located on its exterior surface. Fluorescence spectra of neat BSA and HC/50% BSA co-assemblies are shown in Figure 11, where a significant decrease in the fluorescence intensity and a blue shift of the maximum wavelength from 337 to 326 nm is observed, which demonstrates the hydrophobic environment around tryptophans [71]. Hence, the assumption that hydrophobic interactions excel in these systems is further confirmed. Nonetheless, even though the creation of a protein corona is possible, the HC NPs do not exhibit further aggregation and self-assembled mixed NPs preserve their properties.

### 3.6. Stability Studies

Selected samples of HC–Lyz complexes, CUR-loaded HC NPs, and HC–BSA co-assemblies were investigated by sequential DLS experiments in terms of their size distribution and homogeneity at different time periods. The investigated samples exhibited relative stability for two weeks.

## 4. Conclusions

Novel H-P(MAA-co-LMA) copolymers bearing amphiphilic and polyelectrolyte character combined with distinct hyperbranched macromolecular architecture and topology were efficiently synthesized via a one-step RAFT polymerization reaction. The HCs displayed low CAC values in water media, while their self-assembly properties showed dependence on copolymer hydrophilic–hydrophobic balance, copolymer concentration, and the pH of the aqueous solutions. The self-assembled HC structures were able to solubilize the hydrophobic drug CUR into the core of small-size NPs (mean R_h_ < 10 nm), while the mixed copolymer/drug assemblies exhibited strong fluorescence. Moreover, the protein complexation ability of the HC aggregates was demonstrated by electrostatic complexation of the cationic protein Lyz to the polyanion surface of the formed HC assemblies, whereas co-assembly investigations with the model amphiphilic protein BSA (upon simulation of the physiological media environment) revealed that HC self-assembled structures are stable. Consequently, novel biocompatible P(MAA-co-LMA) HCs could potentially serve as multi-purpose nanocarriers for biorelevant compounds in the field of therapeutics and theranostics.

## Figures and Tables

**Table 1:** Molecular characteristics of the synthesized HCs.

HC	Initial Feed Ratio ^c^	M_w_ ^a^ (g/mol) (×10^4^)	M_w_/M_n_ ^a^	%wt LMA Theoretical	%wt LMA ^b^
HC 1	[40.8]:[6]:[2]:[1]:[0.5]	3.8	1.26	30	41
HC 2	[29]:[9.8]:[2]:[1]:[0.5]	3.6	1.27	50	52

^a^ Determined by SEC in DMF; ^b^ Determined by ^1^H-NMR in THF-d8; ^c^ [MAA]:[LMA]:[EGDMA]:[CTA]:[AIBN] mole ratio.

**Table 2:** DLS and ELS results for HCs in aqueous media in varying pH solutions.

HC Samples	I_90°_ (a.u.)	R_h_ (nm)	PDI	ζ_p_ (mV)	CAC (g mL^−1^)
HC 1 _pH3_	2470	244 (71%)/67 (29%)	0.43	−7.3	--
HC 1 _pH7_	166	40 (92%)/5 (8%)	0.44	−17.8	4.66 × 10^−6^
HC 1 _pH10_	176	33 (90%)/6 (10%)	0.46	−41.8	--
HC 2 _pH3_	16	65	0.66	−6.1	--
HC 2 _pH7_	180	21	0.54	−11.2	4.13 × 10^−6^
HC 2 _pH10_	67	22	0.51	−39.3	--

**Table 3:** DLS and ELS results for HC–Lyz complexes.

HC	HC:Lyz Ratio	C_polymer_ (g/mL)	C_lysozyme_ (g/mL)	I_90°_ (a.u.)	PDI	R_h_ (nm)	ζ_p_ (mV)
1	HC_Non complexed_	1.25 × 10^−4^	-	44	0.48	26 (almost bimodal)	−27.0
1	HC:Lyz = 2:1	1.25 × 10^−4^	9.28 × 10^−4^	2580	0.21	70 (98%)/14 (2%)	+24
1	HC:Lyz = 1.5:1	1.25 × 10^−4^	12.37 × 10^−4^	1355	0.21	87 (99%)/9 (1%)	+20
1	HC:Lyz = 1:1	1.25 × 10^−4^	18.56 × 10^−4^	535	0.25	117 (85%)/38 (14%)	+19
1	HC:Lyz = 1:1.5	1.25 × 10^−4^	27.83 × 10^−4^	104	0.41	94 (95%)/15 (5%)	+25
1	HC:Lyz = 1:2	1.25 × 10^−4^	37.11 × 10^−4^	30	0.65	122 (90%)/6 (10%)	+17
2	HC_Non complexed_	0.75 × 10^−4^	-	3177	0.12	45	−31.1
2	HC:Lyz = 2:1	0.75 × 10^−4^	3.98 × 10^−4^	4250	0.10	55 (97%)/15 (3%)	+31.2
2	HC:Lyz = 1.5:1	0.75 × 10^−4^	5.30 × 10^−4^	4000	0.10	54	+26.8
2	HC:Lyz = 1:1	0.75 × 10^−4^	7.95 × 10^−4^	4500	0.10	55 (91%)/25 (9%)	+35.5
2	HC:Lyz = 1:1.5	0.75 × 10^−4^	11.93 × 10^−4^	4920	0.12	53 (97%)/17 (3%)	+28.2
2	HC:Lyz = 1:2	0.75 × 10^−4^	15.91 × 10^−4^	2968	0.14	48	+25.4

**Table 4:** DLS and ELS results for the neat and CUR-loaded PNPs.

HC Samples	I_90°_ (a.u.)	R_h_ (nm)	PDI	ζ_p_ (mV)
HC 1	105	32	0.43	−33.1
HC 1/10% CUR	125	40	0.48	−35.8
HC 2	93	20	0.50	−27.1
HC 2/10% CUR	110	27	0.50	−38.6

**Table 5:** DLS and ELS results for HC–BSA co-assemblies.

HC Samples	I_90°_ (a.u.)	R_h_ (nm)	PDI	ζ_p_ (mV)
HC 1	155	37 (96%)/3 (4%)	0.43	−7.2
HC 1/20% BSA	162	44 (83%)/7 (17%)	0.49	−11.8
HC 1/50% BSA	149	35 (bimodal)	0.47	−4.5
BSA_Cmin_	20	90 (72%)/4 (28%)	0.50	−19.3
HC 2	135	20	0.50	−7.0
HC 2/20% BSA	205	28	0.49	−4.3
HC 2/50% BSA	210	20	0.51	−8.4

## Data Availability

Data are available on request.

## Supplementary Materials

The following supporting information can be downloaded at: https://www.mdpi.com/article/10.3390/pharmaceutics15041198/s1, Image S1: Photograph of the obtained P(MAA-co-LMA) HCs in solid state; Figure S1: Size distributions of HC 1 (A) and HC 2 (B) in THF and DMF solution at c = 10^−2^ g/mL obtained by DLS measurements; Table S1: DLS results for the HCs in THF and DMF solutions (c = 10^−2^ g/mL); Image S2: Photograph of the CUR loaded HCs in aqueous solutions; Figure S2: ^1^H-NMR spectra of HC 1 (a) and HC 2 (b) in THF-d8 including the corresponding integrals.
