# Supplementary material for: Hyperbranched Copolymers of Methacrylic Acid and Lauryl Methacrylate H-P(MAA-co-LMA): Synthetic Aspects and Interactions with Biorelevant Compounds
Source: Pharmaceutics. 2023 Apr 9;15(4):1198. doi: 10.3390/pharmaceutics15041198 (PMC10140914; doi:10.3390/pharmaceutics15041198)
Supplement: Supplementary file 1 [file pharmaceutics-15-01198-s001.zip › pharmaceutics-2292356-supplementary.pdf]

pharmaceutics

Hyperbranched copolymers of methacrylic acid and lauryl methacrylate H-P(MAA-co-LMA). Synthetic aspects and interactions with biorelevant compounds.

Supplementary Materials

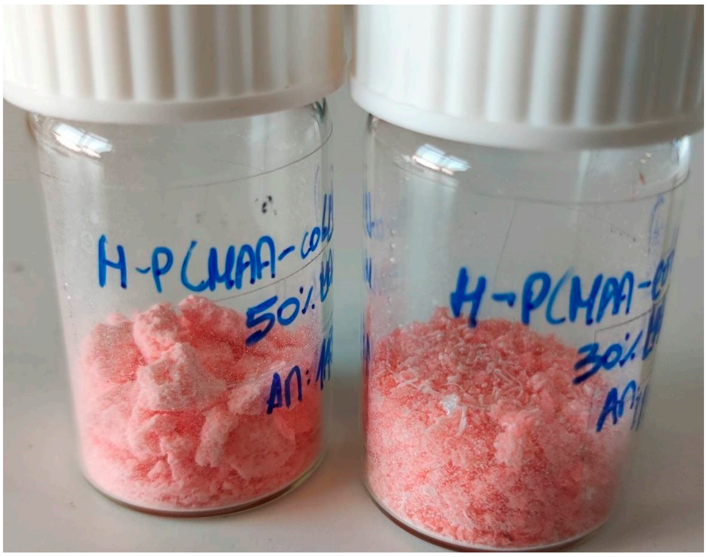

Image S1. Photograph of the obtained P(MAA-co-LMA) HCs in solid state.

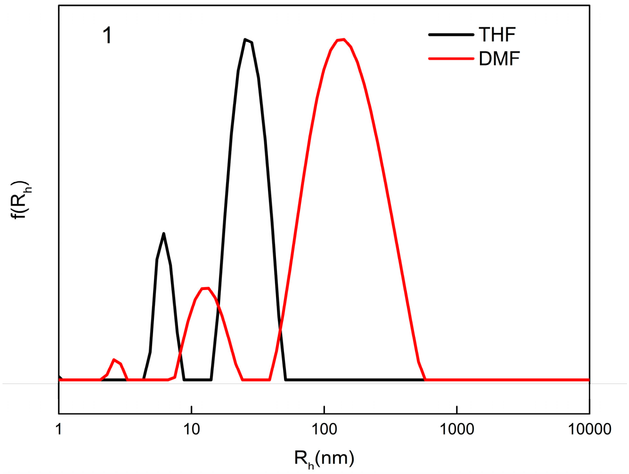

(**A**)

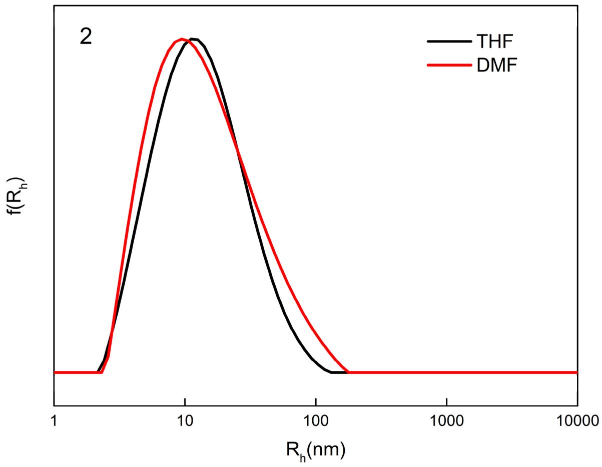

(**B**)

Figure S1. Size distributions of HC 1 (A) and HC 2 (B) in THF and DMF solution at c= 10^{-2}g/mL obtained by DLS measurements.

Table S1. DLS results for the HCs in THF and DMF solutions (c= 10^{-2}g/mL).

HC	$I_{90°}$ (a.u.)	R_h (nm)	PDI
HC 1 in THF	740	27 (83%)/ 6 (17%)	0.39
HC 1 in DMF	1,560	144 (90%)/ 13 (10%)	0.49
HC 2 in THF	253	13	0.38
HC 2 in DMF	175	13	0.38

DLS studies in organic solvents of varying quality for comonomers MAA and LMA were conducted to obtain information about the size of the HCs in the state of free non aggregated chains, if possible. Judging from solvent/monomer polarity THF is expected to be a better solvent for both segments in the HCs while DMF should a better solvent for MAA segments and a bad solvent for LMA segments. Therefore, the results indicate that differences in the solubility of MAA and LMA and composition of the copolymers cause some aggregation of the HCs also in organic solvents. HC 2 presents similar conformation/aggregation behavior in both solvents based on the relatively low scattered intensity and small size. Therefore, the value of 13 nm should be the upper limit for HC 2 single chains in water. On the other hand, aggregation is more evident for HC 1, since very high scattered light intensity values are measured and populations of species in solution are bimodal and polydisperse. Most probably chain aggregation is affected also by hydrogen bonding interactions of MAA segments in the case of HC 1.

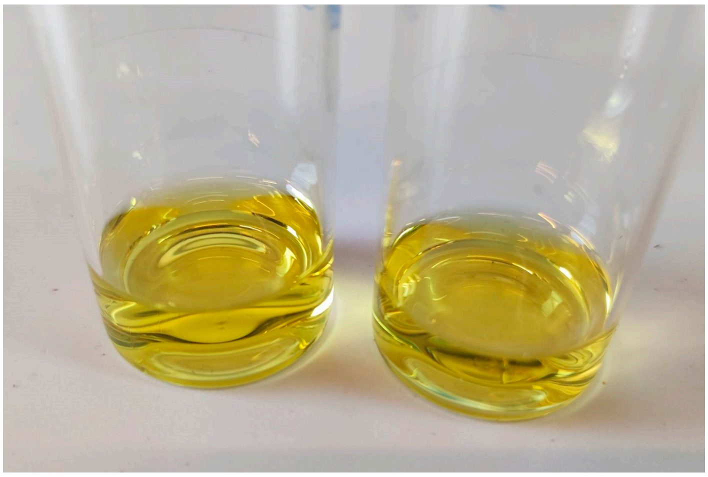

Image S2. Photograph of the CUR loaded HCs in aqueous solutions.

Drug loading content (DLC) and Drug loading efficiency (DLE) by UV-Vis spectroscopy.

DLC and DLE for the CUR loaded PNPs were based on the equations below. DLE was equal to 17.4 % and DLC equal to 1.9% for both copolymers.

$$\text{DLC (wt\%)} = (\text{weight of loaded drug/ weight of drug-loaded polymer}) \times 100, \quad (1)$$

$$\text{DLE (wt\%)} = (\text{weight of loaded drug/ weight of initial drug feed}) \times 100, \quad (2)$$

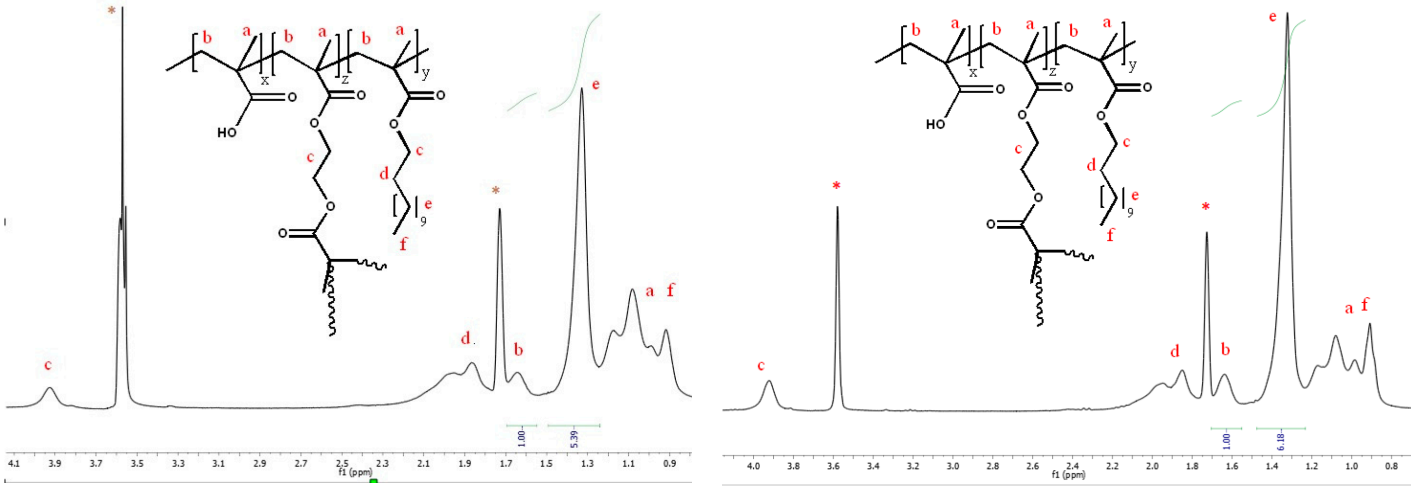

(**A**) (**B**)

Figure S2. ^{1}H-NMR spectra of HC 1 (a) and HC 2 (b) in THF-d8 including the corresponding integrals.
